# Diffusion Weighted Imaging in Unilateral Adrenal Infarction: A Case of Colicky Right Upper Quadrant Pain in a Pregnant Female

**DOI:** 10.7759/cureus.13289

**Published:** 2021-02-11

**Authors:** Rebekah M Padilla, Ashley R Way, Erik Soule, Dheeraj Gopireddy, Chandana Lall

**Affiliations:** 1 Diagnostic Radiology, University of Florida College of Medicine, Jacksonville, USA; 2 Radiology, University of Florida College of Medicine, Jacksonville, USA; 3 Interventional Radiology, University of Florida College of Medicine, Jacksonville, USA; 4 Abdominal Imaging, University of Florida College of Medicine, Jacksonville, USA

**Keywords:** diffusion weighted imaging (dwi), hypercoagulable state, adrenal glands, infarct, apparent diffusion coefficient (adc), right upper quadrant pain, mri imaging, pregnant, antiphospholipid antibody, protein c and protein s deficiencies

## Abstract

Diffusion weighted imaging (DWI) is a magnetic resonance imaging (MRI) non-contrast sequence that can indicate tissue ischemia or infarction. Adrenal infarct may present similarly to biliary or gallbladder pathologies, and the differential diagnosis during emergency work-up can be narrowed utilizing DWI sequences. In this paper, we describe the usefulness of DWI for urgent diagnosis in a case of non-hemorrhagic adrenal infarct of a pregnant female presenting with right upper quadrant pain. Although uncommon, adrenal infarct may occur in patients with hypercoagulability and localizing pain that is unexplained by other imaging modalities. We outline the imaging features of DWI in evaluating adrenal infarct as a safe and time effective application for patients with contraindications to imaging with ionizing radiation.

## Introduction

Diffusion weighted imaging (DWI) is used within specific areas of radiology, such as neuroimaging for stroke. This technique is based on Brownian motion, or the inherent random motion, of a molecule suspended in a fluid. Converse to the idea of random motion, the motion of molecules within cells and organ tissues are somewhat predictable, as certain tissues innately ‘restrict’ the number of diffusion membranes (i.e., molecules within a fluid due to their cellularity). This restricted diffusion is imaged with DWI sequencing by magnetic resonance imaging (MRI), revealing areas of restricted blood flow and possible sites of infarct [1]. These properties make DWI a viable imaging tool since it can predict which organs or pathologic processes restrict molecules (like hemoglobin) within a certain tissue without the use of contrast media or radiation. An apparent diffusion coefficient (ADC) map is then created to verify if an area in question truly restricts diffusion, in order to reduce potential false positives on DWI images [1,2].

The chevron-shaped adrenal glands are located bilaterally, superiomedially, to the kidneys. The normal anatomy of the adrenal gland consists of an outer capsule, cortex (three cortical zones; an outer zona glomerulosa, a middle zona fasciculata, and zona reticularis) and the medulla, found deep to the cortex. Each adrenal gland resides within the suprarenal fat and has a tri-part blood supply. The venous drainage of the right adrenal is facilitated by the right adrenal vein which empties directly into the inferior vena cava (IVC). The left adrenal vein drains to the left renal vein. Due to the anatomy of the adrenal glands and their venous drainage, there is tendency for blood stasis in the adrenal vein, predisposing them to non-hemorrhagic infarction [3].

Virchow’s triad describes clot formation based on blood stasis, endothelial injury, and inflammatory state. Pregnant patients may be at higher risk for venous thrombosis due to hypercoagulability of pregnancy and expected prenatal mass effect within the abdominopelvic cavity on the venous drainage system. It is logical that these two risk factors together, hypercoagulability of pregnancy and adrenal vein anatomy, may exacerbate blood stasis within the adrenal vein and lead to venous thrombosis. The risk of a thrombosis and consequentially infarction should be considered in non-pregnant patients with a known hyper-coagulable state [2-14].

While adrenal hemorrhage is a well-identified adrenal emergency and is reported in patients with underlying infectious etiology and sepsis, non-hemorrhagic adrenal infarct is less common and may be difficult to recognize [2,4,9-12,15,16]. It is estimated that the incidence of non-hemorrhagic adrenal infarct found on MR imaging in pregnant women with abdominal or flank pain is 1.3% [13]. Non-hemorrhagic cases are frequently associated with hypercoagulability which may not be known on initial history or even initial laboratory investigations [9,11,12]. A few of the differentials for localized pain to the right upper quadrant on physical exam include cholelithiasis, cholecystitis, gastritis, and others which may lead to an inappropriate work-up for a surgical diagnosis, particularly in a patient without adrenal insufficiency symptoms. Eliciting the correct diagnosis on imaging is of foremost importance. In our patient’s case, MR imaging and DWI sequences with ADC maps confirmed our patient’s diagnosis of adrenal infarct. In the emergency setting, case studies have described utilizing MRI and DWI sequences with ADC maps of the adrenal glands as a valuable option for identifying adrenal infarcts [2,9,10].

DWI has promising uses outside of neuroimaging and demonstrates applicability for assessing endocrine tissues such as the adrenal glands [3]. MRI of the adrenal glands utilizing DWI and ADC maps provides an alternate, patient-centered approach for diagnosing adrenal infarct particularly in obstetric patients by utilizing nonionizing radiation and avoiding the administration of contrast media. One limitation is DWI reliability for distinguishing between benign and malignant lesions or adenomas and non-adenomas [7,8].

Unfortunately, there is a paucity in literature regarding the protocol of DWI evaluation of the adrenal glands [1,2,4-6]. Our goal is to address the strengths and limitations of DWI imaging sequences of adrenal glands by demonstrating its usefulness in our case and to promote further investigation into this topic and need for standardized adrenal protocol development.

## Case presentation

A 25-year-old pregnant female with a second trimester dichorionic-diamniotic twin pregnancy and past medical history of ulcerative colitis, seizure disorder, anemia, and cholelithiasis presented from an outside hospital with severe colicky right upper quadrant abdominal pain and associated nausea. She had additional past obstetric history of G14P4184, specifically fourteen pregnancies, four live children and multiple fetal losses. Her pain and nausea symptoms were unrelieved with frequent and typical opiate and antiemetic doses. Outside imaging was unavailable, yet, according to the report, there was a negative right upper quadrant ultrasound without signs of cholecystitis.

**Figure 1:**
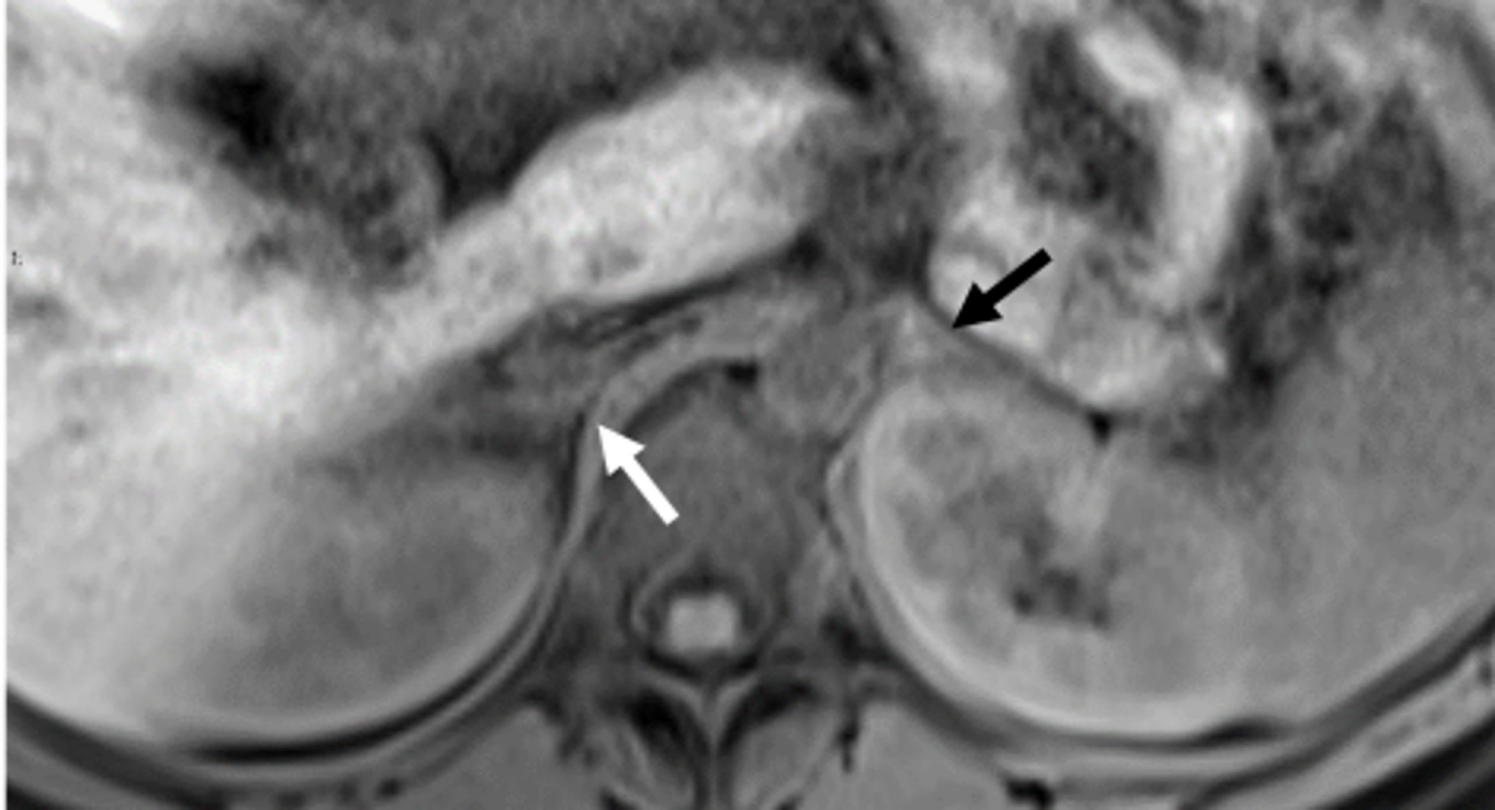
T1-weighted Image at the level of the adrenal glands. T1-weighted fat saturation image showing a diffusely swollen right adrenal gland of intermediate to hypointense signal (white arrow). The left adrenal gland is isointense without edema (black arrow).

A non-contrast magnetic resonance cholangiopancreatography (MRCP) and MRI abdomen/pelvis with DWI sequences were utilized for urgent evaluation of her abdominal pain. MRCP with DWI was negative for biliary tree or gall bladder pathology. On the MRI abdomen exam, as seen in Figure 1, the T1-weighted fat-suppressed image reveals the normal anatomy of the left adrenal gland while the right is diffusely edematous and hypointense.

**Figure 2:**
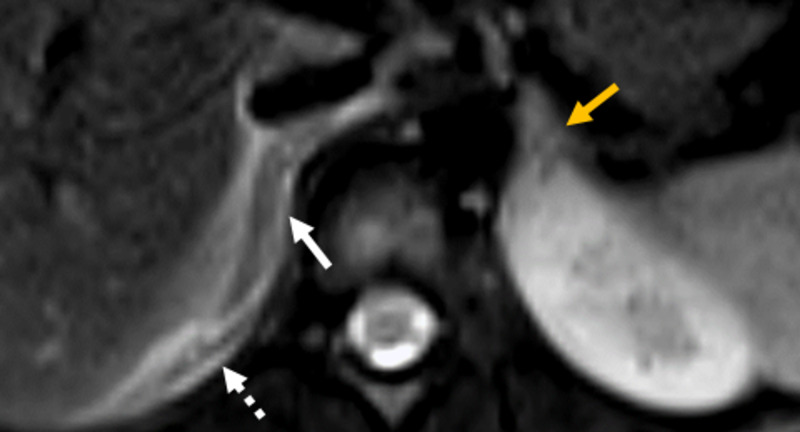
T2-weighted fat-suppressed images. Large, edematous, intermediate signal of the right adrenal gland (white arrow) with periadrenal and perirenal high signal indicating edema (white dashed arrow). The signal intensity of the right adrenal gland is slightly lower than the left. Of note, the left adrenal gland appears normal, without edema (golden arrow).

**Figure 3:**
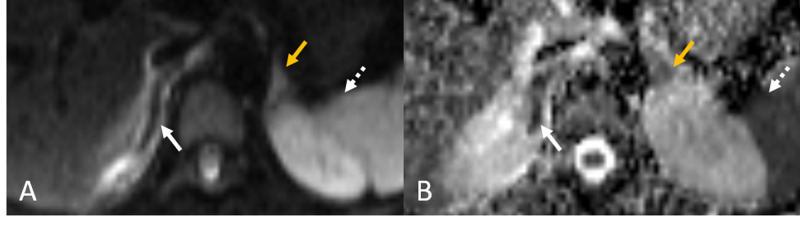
DWI images (A) with corresponding ADC map (B). In Figure A, the left adrenal gland (golden arrow) and spleen (white dashed arrow) show normal bright, hyperintense signal on DWI sequence. To contrast, the infarcted adrenal gland is iso- to hypointense (white arrow). Corresponding restricted diffusion is seen in Figure B, indicated by hypointensity. Restricted diffusion as expected is seen in the left adrenal gland (golden arrow) and spleen (white dashed arrow). Of note, the right adrenal gland (white arrow) restricts diffusion more than the left or the spleen, indicated by its relative hypointensity when compared to the spleen and left adrenal gland on this ADC map. DWI: diffusion weighted imaging, ADC: apparent diffusion coefficient

Correspondingly, in Figure 2, the right adrenal gland is enlarged and edematous with notable periadrenal and perirenal high signal fluid. There is a mixed-signal appearance of the right adrenal gland indicating parenchymal edema. Whereas the adrenal glands are expected to restrict diffusion similar to the spleen or the contralateral adrenal gland, imaging from DWI images show greater than expected diffusion restriction within the right adrenal gland, confirmed with ADC maps (Figures 3A, 3B). B-values of 50 and 500 were used to calculate the ADC map. In comparison, the left adrenal gland and spleen are evaluated with region of interest (ROI) measurements of approximately 1000 units. The infarcted adrenal gland measures at 874 units, indicating greater restricted diffusion due to infarcted parenchyma. The findings of edema, swelling, and greater restricted diffusion were considered diagnostic of right adrenal gland infarct [2,8,9-19].

Our patient’s hospital course followed with a complete hypercoagulability panel, hematology/oncology and endocrinology consultations. She was started on prophylactic low molecular weight heparin. Of note, abnormal laboratory findings included an elevated antineutrophil cytoplasmic antibodies (ANCA) ratio 1:80 and protein C activity of 67%. She improved during her hospital stay with relief of abdominal pain and was discharged on low molecular weight heparin.

## Discussion

The adrenal gland restricts diffusion, to a similar extent, as the spleen. This may be due to the high cellularity of the adrenal gland. The layers of the adrenal gland excrete different hormones, despite the homogenous appearance of the glands and their similar attenuation to nearby muscle on CT and similar intensity to spleen on MR imaging, depending on the sequence. On T1-weighted and T2-weighted imaging the adrenal glands are normally isointense, similar in intensity to the splenic parenchyma. Importantly, the normally functioning adrenal gland restricts diffusion inherently and is hyperintense on DWI and dark on ADC maps. 

Until recently, the role of DWI in the literature indicated value in differentiating adrenal adenomas from malignant lesions and between different lipid content of adenomas [7,8]. According to Tsushima et al., DWI does not show a significant difference between adrenal adenoma and metastatic tumors but does illustrate an ADC value of adenomas significantly lower than pheochromocytomas [7]. Similarly, Miller et al. describe a large range of overlap between adrenal metastasis and adenomas as well as poor differentiation between lipid poor and lipid-rich adenomas on DWI and ADC maps [8]. The normal adrenal glands show no significant difference in signal intensity between the right and left glands.

Upon infarction, the adrenal gland typically becomes swollen and periadrenal edema develops. On plain MR T1-weighted imaging, hyperintense signal around the adrenal gland may be high, appearing bright, or hyperintense. Depending on the acuteness of infarct, the affected adrenal gland may appear hyperintense on T1-weighted imaging. For instance, a case report of ischemic thrombosis describes a T2 hypointense adrenal infarct early and a T2 hyperintense infarct in a sub-acute setting due to blood product conversion to methemoglobin [10]. Swelling, edema, and change of adrenal gland signal are signs of infarction that we believe can be confirmed with diffusion imaging [2-5,13,15]. Molière et al. describe a case of bilateral adrenal infarction which showed hyperintense to heterogeneous signal on DWI and a corresponding low signal on ADC maps [14]. Similarly, on DWI images we see a hyperintense to isointense adrenal gland and on corresponding ADC maps a strongly hypointense, restriction of diffusion. Of note, the infarcted adrenal gland in Figure 3A, 3B appears to restrict diffusion more than the contralateral normal adrenal gland and more than the spleen ROI average intensity. This is the key imaging finding to determine adrenal infarct. This allows a diagnosis without using contrast media.

Interestingly, the adrenal infarct on DWI images is hypointense compared to what we would expect to see for an infarcted gland. We suspect the infarct could cause very small parenchymal micro-hemorrhages and these small hemorrhages cause a hypointense, low signal on DWI [1]. Another possibility is the hypointense low signal on DWI is due to the degradation of blood products to methemoglobin as explained by Berneis et al. [10]. However, susceptibility sensitive sequences were negative for susceptibility artifact, and therefore blood products, ruling out hemorrhage.

There are limitations when examining DWI imaging in cases of infarction. Specifically, with low B values, the ability of DWI and ADC maps to differentiate a perfusion deficit versus a diffusion restriction comes into question [20]. It is possible in our case that a perfusion limitation caused by lack of venous drainage and ultimately impaired organ perfusion affected the apparent restricted diffusion as seen on ADC maps [3,20]. The radiological distinction of diffusion restriction and perfusion defect likely approximate each other in a state of infarction due to pathological fluid stasis. This may explain why DWI values are isointense.

Based on our observations and other similar cases, Table 1 shows the expected intensity of normal, infarcted, and hemorrhagic glands on typical MR imaging sequences [1,7,8,10,12,14,17-19].

**Table 1:** Standard intensity of the normal, ischemic, and hemorrhagic adrenal gland Key: diffusion weighted intensity (DWI), apparent diffusion coefficient (ADC), fat-saturated (FS) ^1^Compared to typical adrenal diffusion restriction and splenic diffusion restriction. Acute < 7 days, Subacute: 7-10 days Hypointense (Dark gray), Isointense (Gray), Hyperintense (White)

Adrenal gland	T1-weighted	T1-weighted FS	T2-weighted	T2-weighted FS	DWI	ADC map
Normal	Isointense	Isointense to Hypointense	Isointense	Hypointense	Hyperintense	Hypointense
Infarction	Isointense acute, Hyperintense subacute	Isointense	Hypointense acute, Hyperintense subacute	Hypointense	Isointense to Hyperintense	Significantly Hypointense^1^
Hemorrhage	Hyperintense	Hyperintense	Hyperintense, slightly	Hyperintense, slightly	Hyperintense	Hypointense

## Conclusions

In a patient such as a child or pregnant female, utilizing imaging with low or no ionizing radiation should be of high priority while studies with moderate to high doses of radiation such as CT or nuclear medicine should be reserved for emergent or life-threatening situations. In our case, MRI with DWI and ADC maps was used to confirm adrenal infarction and adapt the management for a patient who may have otherwise undergone emergency surgery.

DWI in conjunction with ADC maps shows promise in diagnosis of adrenal infarction. Adrenal infarction may not be the leading differential diagnosis in patients with localizing abdominal or flank pain; however, we believe it should be highly considered in pregnant females especially with a history of multiple failed pregnancies. The capability and effectiveness of DWI and ADC maps to diagnose adrenal infarct need further study and research. DWI with ADC maps is a promising and viable tool to diagnose non-hemorrhagic adrenal infarct.
